# Soil organic matter rather than ectomycorrhizal diversity is related to urban tree health

**DOI:** 10.1371/journal.pone.0225714

**Published:** 2019-11-22

**Authors:** Maarten Van Geel, Kang Yu, Gerrit Peeters, Kasper van Acker, Miguel Ramos, Cindy Serafim, Pierre Kastendeuch, Georges Najjar, Thierry Ameglio, Jérôme Ngao, Marc Saudreau, Paula Castro, Ben Somers, Olivier Honnay

**Affiliations:** 1 Plant Conservation and Population Biology, Department of Biology, KU Leuven, Kasteelpark Arenberg, Heverlee, Belgium; 2 Division of Forest, Nature & Landscape, Department of Earth & Environmental Sciences, KU Leuven, Celestijnenlaan, Heverlee, Belgium; 3 Escola Superior de Biotecnologia, Catholic University of Portugal, Rua Arquiteto Lobão Vital, Porto, Portugal; 4 Laboratoire des Sciences de L'ingénieur, de L'informatique et de L'imagerie, Strasbourg University, Illkirch, France; 5 Université Clermont Auvergne, INRA, PIAF, Clermont Ferrand, France; Trent University, CANADA

## Abstract

Urban trees provide many ecosystem services, including carbon sequestration, air quality improvement, storm water attenuation and energy conservation, to people living in cities. Provisioning of ecosystem services by urban trees, however, may be jeopardized by the typically poor quality of the soils in urban areas. Given their well-known multifunctional role in forest ecosystems, ectomycorrhizal fungi (EcM) may also contribute to urban tree health and thus ecosystem service provisioning. Yet, no studies so far have directly related *in situ* EcM community composition to urban tree health indicators. Here, two previously collected datasets were combined: i) tree health data of 175 *Tilia tomentosa* trees from three European cities (Leuven, Strasbourg and Porto) estimated using a range of reflectance, chlorophyll fluorescence and physical leaf indicators, and ii) ectomycorrhizal diversity of these trees as characterized by next-generation sequencing. Tree health indicators were related to soil characteristics and EcM diversity using canonical redundancy analysis. Soil organic matter significantly explained variation in tree health indicators whereas no significant relation between mycorrhizal diversity variables and the tree health indicators was found. We conclude that mainly soil organic matter, through promoting soil aggregate formation and porosity, and thus indirectly tree water availability, positively affects the health of trees in urban areas. Our results suggest that urban planners should not overlook the importance of soil quality and its water holding capacity for the health of urban trees and potentially also for the ecosystem services they deliver. Further research should also study other soil microbiota which may independently, or in interaction with ectomycorrhiza, mediate tree performance in urban settings.

## Introduction

Urban areas are heavily modified anthropogenic ecosystems and within the next decade, more than half the world’s population will be living in urban areas [1]. However, environmental problems, such as air pollution, increased temperatures (heat island effect), noise and flooding, jeopardize health and quality of life of the urban population [2–4]. Urban trees play a crucial role in mitigating these environmental problems as they provide many ecosystem services (reviewed in [5]). They can sequester carbon, improve air quality, alleviate flooding events and modify the microclimate [5,6]. Urban trees can also reduce energy consumption [7], crime [8], mental distress [9,10], and enhance urban aesthetics and property value [11]. Urban soil conditions, however, may negatively affect urban tree health and jeopardize the delivery of their ecosystem services. Urban trees commonly grow in soils sealed by buildings and urban infrastructures and are limited by a lack of rooting space and nutrient holding capacity [12]. They are also more prone to water deficits than forest trees, making them susceptible to pathogens and pests [13,14]. These limitations can compromise urban tree health and reduce tree survival and life expectancy, jeopardizing the ecosystem services delivered by urban trees [14,15]. Although the relation between tree health and soil conditions in natural forests has been extensively studied (reviewed in [16]), the relation between soil conditions and tree health in urban areas has received relatively little attention.

Another important driver of urban tree health may be the presence of ectomycorrhizal fungi (EcM). In natural temperate and boreal forests, most tree species form a symbiosis with various EcM. Through their extraradical hyphal network, EcM provide a range of benefits to their host [17]. In return for plant photosynthates, the hyphal network extends the root system, increasing uptake of water and nutrients [18,19]. Moreover, EcM can stimulate the plant immune system (mycorrhiza-induced resistance), protecting the host against a wide range of attackers (nematodes, herbivorous arthropods and pathogens) [20]. The fungal mantle surrounding the roots can also physically prevent pathogens from penetrating the roots [21]. Because of these benefits, EcM can improve tree health, and thus the extent and resilience of ecosystem services delivered by urban trees.

EcM are diverse and functionally different, for example regarding the formation of extra-radical hyphae, colonization rates and their phosphorus foraging strategy [22,23]. Many EcM can also simultaneously colonize tree roots [24,25]. Although the effect of environmental factors on EcM communities have been well investigated (e.g. [24,26]), the functional role of EcM diversity on tree health is largely unexplored (but see [27,28]). Diverse communities of EcM may promote tree health through a few highly productive EcM taxa with a large influence on the host, and through functional complementarity among EcM taxa, increasing the total resource capture [29,30]. Baxter and Dighton [31], for example, showed that EcM diversity increased root biomass and phosphorus uptake of *Betula populifolia* seedlings. Additionally, Maherali and Klironomos [32] showed that functional complementarity among distant lineages of arbuscular mycorrhizal taxa reduced competition and enhanced plant productivity. Therefore, EcM communities with high phylogenetic diversity may better promote tree health compared to fungal communities with low phylogenetic diversity. Although the potential role of mycorrhizal (phylogenetic) diversity on the host plant has been intensively studied in greenhouse experiments, it is still unclear whether benefits from mycorrhizal diversity are realized *in situ*, where abiotic conditions fluctuate and the host simultaneously interacts with a range of biota [33,34].

The objective of this study was to understand to what extent soil characteristics and mycorrhizal diversity can explain variation in urban tree health. Tree health status of 175 urban trees from three European cities, namely Leuven (Belgium), Strasbourg (France) and Porto (Portugal) was estimated using a range of reflectance, chlorophyll fluorescence and physical leaf indicators. Ectomycorrhizal fungal diversity of these trees was characterized by next-generation sequencing. We specifically aimed to disentangle the relative importance of general soil characteristics and ectomycorrhizal (phylogenetic) diversity on tree health in urban areas. Our hypothesis was that i) organic matter and nutrient content of the soil and ii) EcM diversity are positively related to urban tree health.

## Material and methods

This study combines tree health data obtained by Yu et al. [35] with soil and ectomycorrhizal diversity data obtained by Van Geel et al. [24] (S1 Table). Below is summarized how these data were collected. For all the details, we refer to Yu et al. [35] and Van Geel et al. [24].

### Study sites and sampling

Urban trees were sampled in three European cities: Leuven (Belgium; 50°52'36"N, 4°42'16"E), Strasbourg (France; 48°34'00"N, 7°45'33"E) and Porto (Portugal; 41°09'20"N 8°37'44"W). *Tilia tomentosa* was chosen as the model species because of its dependency on EcM and its common use for landscaping in European urban areas and monitoring of urban environmental quality [36,37]. Across the three cities, 175 *T*. *tomentosa* trees (Leuven N = 52; Strasbourg N = 56; Porto N = 67) were randomly selected from a city map indicating all the urban trees. First, sites in the city were selected to obtain a representative sampling across the whole city. Within one site, trees were arbitrarily selected for sampling. The greenery service of each city issued the permission to sample the selected trees (i.e. for Leuven: *Groendienst Leuven*; Strasbourg: *Eurometropole de Strasbourg*, *Service espaces verts et de nature*; Porto: *Câmara Municipal do Porto*). Selected trees were sampled in September-October 2017. For each tree, three arbitrarily chosen branches located respectively within three equally sized sectors (i.e. nadir view) of the crown were cut and of each branch five intact leaves were collected (i.e. 15 leaves per tree). For each tree, three arbitrarily chosen roots, approximately 1–2 m from the stem and located within three equally sized sectors around the stem (i.e. nadir view) were excavated. These three root samples were pooled to obtain one pooled root sample per tree. Next to each root sample, also a soil core (10 cm depth, 3.5 cm diameter) was collected and the three cores were pooled to obtain one pooled soil sample per tree. In total, 15 × 175 leaf, 175 root and 175 soil samples were obtained across the three cities. Tree diameter at breast height (DBH) was determined by measuring the trunk diameter at 1.40 m height.

### Tree health indicators

Tree health status was estimated using a range of reflectance, chlorophyll fluorescence and physical leaf indicators (Table 1). First, reflectance of the leaves was measured using an ASD FieldSpec 3 spectroradiometer (ASD, Longmont, CO, USA) connected to a Plant Probe combined with a Leaf Clip Assembly (ASD). The spectroradiometer provides a 3 nm spectral resolution and measures reflectance ranging from 350–2500 nm. The upper side of the leaf was measured on three random spots (of 10 mm). The 45 spectra obtained for the 15 leaf samples were averaged per tree. Using these spectra, the following spectral indices were determined indicative for tree health and related to (i) plant chlorophyll content—mSR705 and mND705 [38], (ii) water status—NDWI, MDWI, WI and WI2 [39–42], (iii) photosynthetic light use efficiency—PRI [43], and (iv) plant senescence—PSRI and SIPI [44,45]. Next, chlorophyll fluorescence, which estimates photosynthetic efficiency, of each sampled leaf was measured after dark adaptation of 25 minutes using a Handy PEA fluorometer (Hansatech Instruments, Pentney, UK) to obtain the *maximum efficiency of PSII* (Fv/Fm) and *performance index* (PI) indicators [46]. The 15 measurements of each leaf were averaged per tree. Fv/Fm reveals changes in a wide range of plant stresses and PI indicates plant vitality [46,47]. Finally, leaf water content (LWC) of the leaves was determined by the weight loss at 75°C and calculated as the ratio of water content and fresh weight. Leaf water per area (LWA) was calculated as the ratio of water content and leaf area, and specific leaf area (SLA) as the ratio of leaf area and dry weight.

**Table 1:** Reflectance, chlorophyll fluorescence and physical leaf indicators used to estimate tree health. R_x_ = reflectance at wavelength x, F_v_ = variable fluoresence, F_m_ = maximum fluorescence, F_0_ = fluorescence at 50 μs, M_0_ = initial slope of fluorescence, and V_j_ = relative variable fluorescence at 2 ms.

Tree health indicator	Description [reference]	Formula	Range (min, max, mean)
*Reflectance indicators*			
mSR705	Modified simple ratio using wavelength at 705 nm [38]	(R_750_ – R_445_)/(R_705_ – R_445_)	1.3, 6.3, 4.1
mND705	Modified NDVI using wavelength at 705 nm [38]	(R_750_ – R_705_)/(R_750_ + R_705_ - 2R_445_)	0.1, 0.7, 0.6
NDWI	Normalized difference water index [40]	(R_860_ – R_1240_)/(R_860_ + R_1240_)	-0.03, 0.03, 0.02
MDWI	Maximum difference water index [39]	(Rmax_1500~1750_ – Rmin_1500~1750_)/(Rmax_1500~1750_ + Rmin_1500~1750_)	0.1, 0.3, 0.2
WI	Water index [41]	R_900_/R_970_	0.97, 1.02, 0.98
WI2	Water index using SWIR bands [42]	R_1300_/R_1450_	1.4, 3.3, 2.69
PRI	Physiological reflectance index [43]	(R_531_ – R_570_)/(R_531_ + R_570_)	-0.2, 0.1, 0.03
PSRI	Plant senescence reflectance index [44]	(R_680_ – R_500_) /R_750_	0.0, 0.4, 0.01
SIPI	Structure insensitive pigment index [45]	(R_800_ – R_445_)/(R_800_ – R_680_)	1.0, 1.8, 1.02
*Fluorescence indicators*			
Fv/Fm	Maximum efficiency of photosystem II [46]	F_v_/F_m_	0.1, 0.8, 0.81
PI	Performance index [46]	((1 –(F_0_/F_m_))/(M_0_/V_j_)) × ((F_m_-F_0_)/F_0_) × ((1-V_j_)/V_j_)	1.2, 10.8, 4.59
*Physical indicators*			
LWC	Leaf water content	water content/fresh weight	38.6, 67.0, 57.3
LWA	Leaf water per area	water content/leaf area	2.7, 14.1, 9.91
SLA	Specific leaf area	leaf area/dry weight	9.4, 29.9, 14.23

### Soil chemical analysis

For each soil sample, pH was measured using a glass electrode in a 1:10 soil/water mixture. As a measure of plant-available P content of the soil, Olsen P was quantified by shaking 2 g of dry soil for 30 minutes with 0.5 M sodium bicarbonate at pH 8.5 and subsequent colorimetric analysis using the molybdenum blue method [48]. As a measure of plant-available N content of the soil, ammonium and nitrate were quantified by shaking 10 g of soil in 200 mL of 1 M potassium chloride solution for one hour and colorimetric analysis of using the salicylate method for ammonium and the N-(1-Naphthyl)ethylenediamine method for nitrate [48]. Extracts were colorimetrically analyzed using the Evolution 201 UV-visible Spectrophotometer (Thermo Scientific, Waltham, MA, USA). Moisture content was measured by the weight loss of 10 g of fresh soil after evaporation of moisture at 105°C. Organic matter was determined by weight loss of 5 g of dry soil after combustion of organic matter at 700°C.

### Measuring ectomycorrhizal diversity using next generation amplicon sequencing

DNA from the root samples was extracted from 100 mg root material using the Soil DNA Isolation Kit (Norgen, Ontario, Canada). All root DNA extracts were amplified by PCR targeting the ITS2 region of the rRNA gene using the sample-specific barcode-labelled versions of the primers ITS86F and ITS4 [49]; dual-index sequencing strategy). PCR was performed on a Bio-Rad T100 thermal cycler (Bio-Rad Laboratories, CA, USA) in a reaction volume of 25 μl, containing 0.25 mM of each dNTP, 0.5 μM of each primer, 1× HiFi Buffer, 1U HiFi DNA Polymerase (HighQu, Kraichtal, Germany), and 1 μl genomic DNA. DNA samples were denatured at 95°C for 60 s. Next, 35 cycles were ran, consisting of 20 s at 95°C, 30 s at 52°C and 30 s at 72°C. Amplicons were purified using the Agencourt AMPure XP kit (Beckman Coulter Life Sciences, Indianapolis, IN, USA) and subsequently quantified using the Quant-iT PicoGreen dsDNA Assay Kit and Qubit fluorometer (Invitrogen, Ghent, Belgium). The amplicons were pooled in equimolar quantities (2 nM) and sequenced using an Illumina MiSeq platform with v2 500 cycle reagent kit (Illumina, San Diego, CA, USA).

Sequences from the Illumina run were clustered into operational taxonomic units (OTUs) using USEARCH following the recommended pipeline [50]. First, paired-end reads were merged to consensus sequences. Consensus sequences were filtered allowing a maximum expected error of 0.5. Next, sequences were clustered into OTUs defined at 97% sequence similarity using the UPARSE algorithm, during which chimeric sequences were also removed [50]. OTUs were taxonomically identified by querying the representative sequence against the UNITE ITS database [51] using the SINTAX algorithm [52]. OTUs were parsed into ecological guilds using FUNGuild [53]. Only ectomycorrhizal OTUs were retained in the dataset. To prevent bias due to different sequencing depth, reads were randomly resampled to 866 reads per sample. In this way, the number of reads and samples retaining in the dataset was maximized. 25 samples with fewer than 866 reads were omitted, leaving 150 trees in the dataset.

Several measures of ectomycorrhizal diversity measures were calculated. First, ectomycorrhizal richness was determined as the number of ectomycorrhizal OTUs present in a sample. Next, Shannon diversity (H) was calculated using the *diversity* function of the R-package *vegan* [54]. Shannon diversity was exponentially transformed (Exp(H)) to linearize the variable (see Jost [55] for details). To account for the phylogenetic relation between EcM OTUs, the most common phylogenetic diversity index was determined, i.e. Faith’s phylogenetic diversity [56]. It incorporates phylogenetic differences and is calculated as the sum of the branch lengths in a phylogenetic tree of the members in a community. Therefore, a maximum likelihood phylogenetic tree using the representative sequences of the EcM OTUs was constructed based on a *Muscle* alignment in Mega X [57,58]. Based on this phylogenetic tree and the OTU-sample dataset, Faith’s phylogenetic diversity was calculated using the *pd*.*query* function of the R-package *PhyloMeasures* [59]. This metric was also standardized for variation in OTU richness using the abundance-weighted null model with 9999 Monte-Carlo randomizations. The total number of reads per OTU were used as abundance weight. In this way, more abundant OTUs were more likely to occur in the Monte-Carlo randomizations.

### Statistical analysis

To investigate the effect of soil chemical variables (soil pH, Olsen P, total N, moisture content and organic matter) and EcM diversity (EcM richness, Exp(H), Faith PD and Faith PD standardized) on tree health indicators (mSR705, mND705, NDWI, MDWI, WI, WI2, PRI, PSRI, SIPI, Fv/Fm, PI, LWC, LWA and SLA), a canonical redundancy analysis (RDA) was performed in the R-package *vegan* [54]. RDA is a direct gradient analysis technique that summarizes linear relationships between components of the response variables that are explained by a set of explanatory variables. It thus allows studying the relationship between a multivariate response matrix (i.e. the tree health indicators) on the one hand, and an explanatory matrix (i.e. soil and EcM variables) on the other hand. To account for differences between cities and diameter at breast height, these variables were also included in the explanatory matrix. Before RDA, all tree health indicators were standardized using the *decostand* function (R-package *vegan*). To check for collinearity among explanatory variables, we calculated variation inflation factors (VIF). All VIF values were lower than five indicating there was no problematic amount of collinearity. To identify the explanatory variables that significantly explained variation in tree health indicators, forward selection was performed using the *ordistep* function (R-package *vegan*) (1000 Monte Carlo permutations, α < 0.05). To test the significance of the selected variables in the model, permutation tests were performed on the individual terms (1000 permutations) using the *anova*.*cca* function (R-package *vegan*). When city or diameter at breast height were selected by the forward selection procedure, the variation attributed to these variables was accounted for using the *Condition* statement in the final RDA model, i.e. a partial RDA accounting for city or diameter at breast height was performed. The main scope of this study was to understand to what extent soil characteristics and mycorrhizal diversity can explain urban tree health, not to test whether tree health indicators vary across cities, or change with varying diameter at breast height. Therefore, the main explanatory variables in the RDA model were the soil chemical and mycorrhizal diversity variables, and a partial RDA was used to account for the effects of city and diameter at breast height. Furthermore, relations between soil moisture content and other soil variables were explored using mixed models (with city as a random factor) in JMP pro (v. 14).

As a complementary analysis to RDA, variance partitioning was performed using the *varpart* function (R-package *vegan* [60]). Variance partitioning allows to investigate the unique contribution of an explanatory matrix while accounting for other explanatory matrices to explain the total variation in the response matrix. Here, three explanatory data matrices were used: (i) soil data, (ii) ectomycorrhizal diversity and (iii) city (to account for differences between cities). Therefore, variance partitioning allows to test for the unique effect of ectomycorrhizal diversity on the variation in tree health indicators, while accounting for soil variables and differences between cities. Conversely, it allows to test for the unique effect of the soil chemical data on the variation in tree health indicators, while accounting for ectomycorrhizal diversity and differences between cities. Only the significant explanatory variables, as determined by forward selection (*ordistep* function), in each of the three matrices were included in the variance partitioning.

## Results

### Canonical redundancy analysis

**Fig 1:**
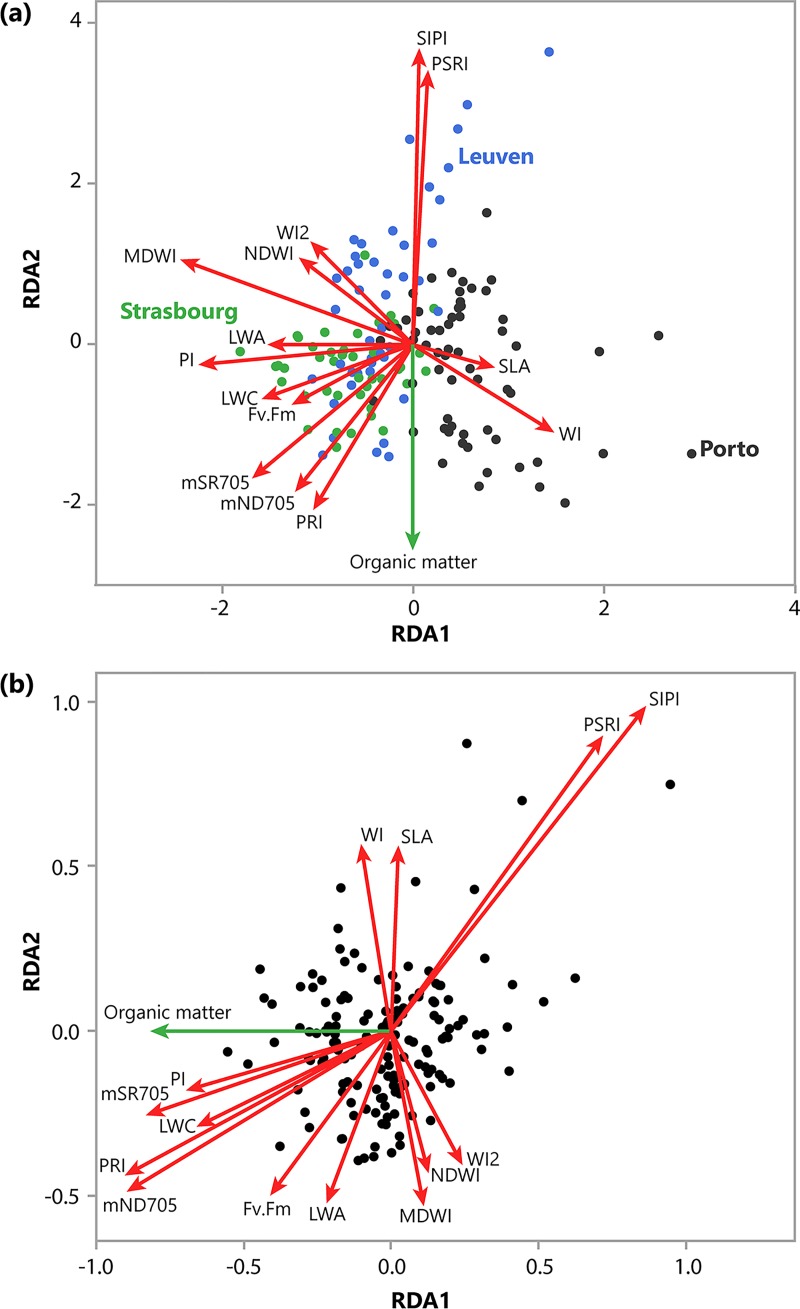
**Redundancy analysis (RDA) triplots of the tree health indicators (N = 150) across Leuven (blue), Strasbourg (green) and Porto (red) with city in the model (a) and the variation of city accounted for (partial RDA) (b).** Green arrows indicate environmental variables explaining a significant proportion of the tree health indicators (as determined with forward selection), and red arrows indicate tree health indicators (response variables). Arrows point out the direction of the increasing gradient in the ordination space. The angles between arrows approximate the correlation between response and environmental variables. Abbreviations, description, reference and formulas used to calculate the tree health indicators are presented in Table 1.

**Fig 2:**
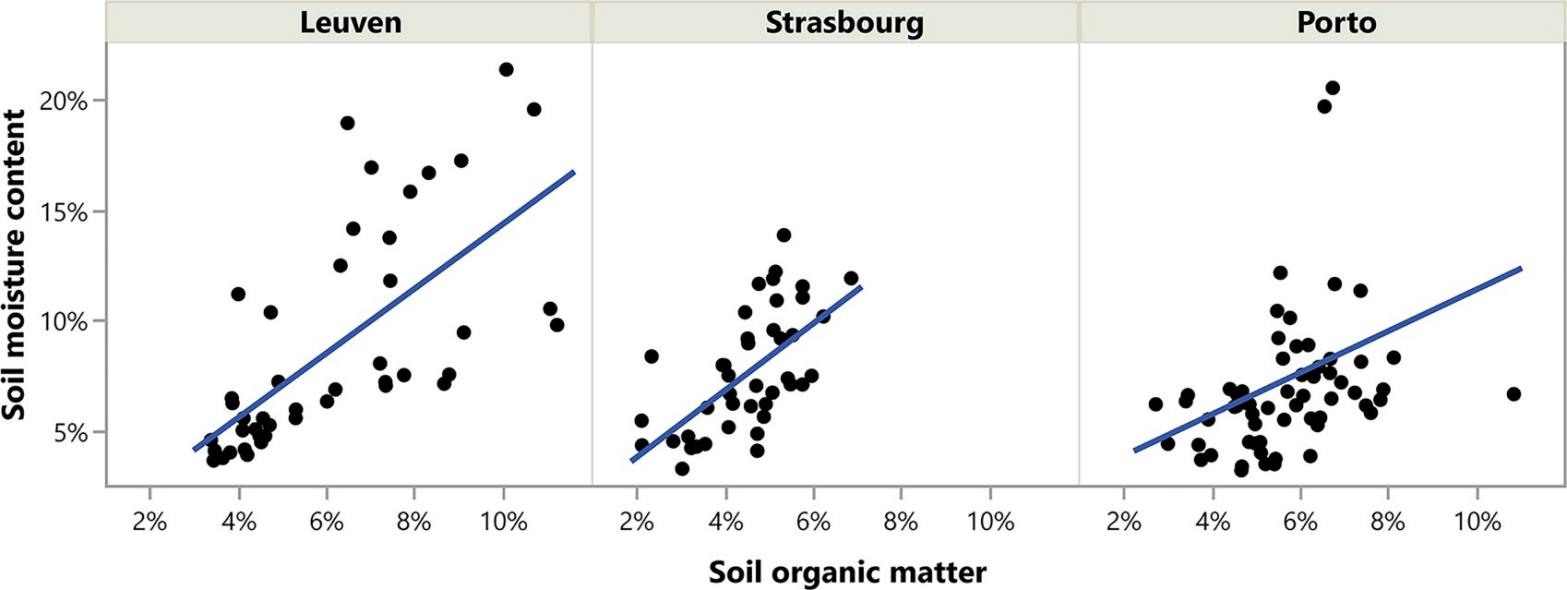
The relation between soil moisture content and soil organic matter in the three sampled cities. A mixed model with moisture content as response variable and soil organic matter as explanatory variable (and city as random factor) showed a strong relation between soil organic matter and soil moisture content (*F* = 53.7, *P* < 0.001).

RDA with forward selection selected city and soil organic matter content, and not any of the EcM diversity variables, as the explanatory variables significantly explaining variation in tree health indicators (Fig 1). The final model explained 18.7% of the total variation in tree health indicators. RDA thus revealed differences in tree health indicators between cities (*F* = 15.85, *P* < 0.001)(Fig 1A) and, after accounting for differences between cities, a strong relation between the tree health indicators and organic matter (*F* = 5.76, *P* = 0.003) (Fig 1B). As city was not a variable of interest, the variation attributed to this variable was accounted for using a partial RDA (Fig 1B). The small angle between organic matter in the soil and PI, mSR705, LWC, PRI and mND705 tree health indicators indicates a high correlation between these variables (Fig 1B). Conversely, the angle between organic matter and PSRI and SIPI health indicators indicates a negative correlation between these variables. The angle between organic matter and WI, SLA, Fv.Fm, LWA, MDWI, NDWI and WI2 approximates 90°, indicating no correlation between these variables. The RDA did not reveal a significant effect of EcM diversity variables on the tree health indicators. Furthermore, the mixed models revealed that soil organic matter was the only variable that was significantly related to moisture content in the soil (*F* = 53.7, *P* < 0.001; Fig 2).

### Variation partitioning

**Fig 3:**
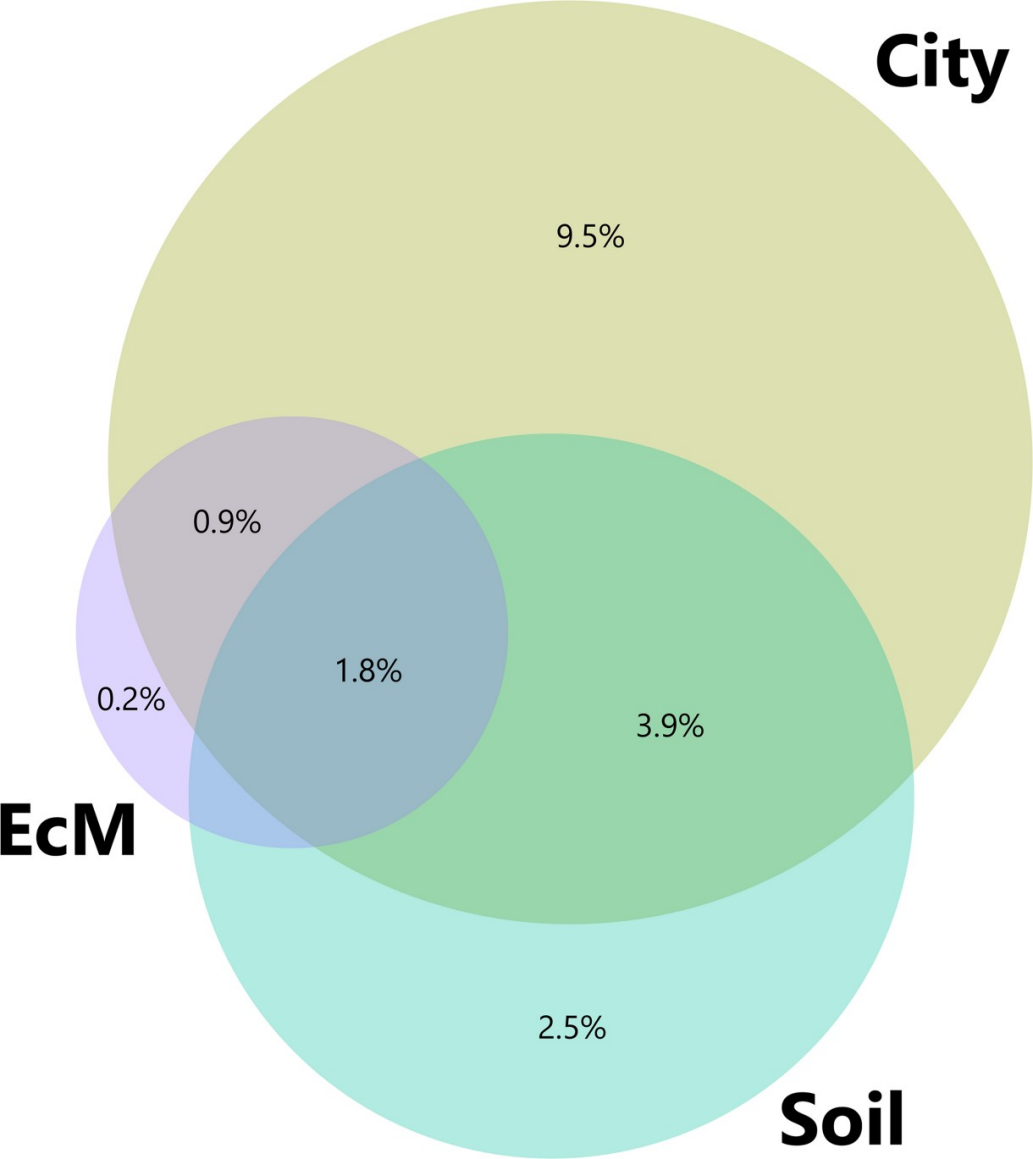
Venn diagram representing variance partitioning results of tree health indicators among three explanatory matrices, i.e. EcM diversity variables, soil chemical variables and the variable city. The size of the circles is proportional to the variability in tree health indicators as explained by a particular explanatory matrix, while overlap of the circles represents the shared variation among explanatory matrices. Numbers indicate the adjusted R^2^ values and thus the variability explained by each partition.

The variable city explained significant variation of the tree health indicators (*F* = 15.357, *P* < 0.001). Among the soil variables, the forward selection procedure selected pH (*F* = 13.725, *P* < 0.001) and organic matter (*F* = 4.407, *P* = 0.010) as significantly related to the tree health indicators. Among the EcM diversity variables, Faith phylogenetic diversity was the only variable selected to explain significant variation in tree health indicators (*F* = 6.144, *P* < 0.001). Comparison of the three different explanatory matrices using variance partitioning revealed that the variable city explained the most unique variation in tree health indicators (R^2^ adjusted = 9.5%) (Fig 3). The chemical soil variables explained a significant portion of the variation (R^2^ adjusted = 2.5%), while the EcM diversity explained little to no unique variation (R^2^ adjusted = 0.2%) in tree health indicators (Fig 3). Indeed, a partial RDA accounting for the variable city and the soil variables revealed no significant effect of EcM diversity on the tree health indicators (*F* = 1.006, *P* = 0.385). A large part of the variation explained by the soil chemical variables was shared with the variable city (R^2^ adjusted = 3.9%).

## Discussion

To our knowledge, this study is the first to combine soil characteristics and ectomycorrhizal diversity data to explain tree health in urban areas. Our results reveal that soil organic matter rather than mycorrhizal diversity explained variation in urban tree health indicators.

### Soil organic matter and tree health

Soil organic matter was the only soil variable that was related to tree health. Our analysis revealed that soil organic matter coincided with leaf water content, indicating that urban trees growing in soils with higher organic matter experienced less water stress. Soil organic matter was also related to the chlorophyll content in the leaves, photosynthetic light use efficiency and the spectral performance index, indicating better photosynthetic capacity and hence tree health and growth. It has been shown that soil organic matter strongly correlates with the volume of available soil water, as soil organic matter is hydrophilic in nature and promotes soil aggregate formation and the development of soil porosity that enhances infiltration and retention of water (reviewed in [61,62]). Our results also revealed a strong relation between organic matter and moisture content in the soil, which may explain the relation between soil organic matter and tree water availability, photosynthetic capacity and vitality.

### Ectomycorrhizal diversity does not explain tree health

To test whether mycorrhizal diversity positively affected tree health status in urban areas, several mycorrhizal diversity measures based on next-generation amplicon sequencing were related to tree health. If mycorrhizal diversity in the roots of urban trees would positively affect tree health, mycorrhizal diversity measures would explain significant variation in the reflectance, chlorophyll fluorescence and physical leaf tree health indicators. Our analysis, however, revealed that, when accounting for soil variables, no significant variation in tree health indicators could be explained by ectomycorrhizal richness or Shannon diversity. It was also expected that mycorrhizal fungal communities with high phylogenetic diversity, i.e. having more distant mycorrhizal lineages that complementing each other, would better promote tree health in comparison to low phylogenetic diversity. Yet, our analysis also revealed that both Faith’s phylogenetic diversity and Faith’s standardized phylogenetic diversity did not significantly explain variation in tree health indicators.

Although we used state of the art methods to assess tree health and mycorrhizal diversity, several methodological limitations may have prevented the observation of positive effects of mycorrhizal diversity on tree health. Ironically, one of the main motivations of this study, i.e. to show whether benefits from mycorrhizal diversity are realized *in situ*, may be the reason no significant effect of mycorrhizal diversity on tree health was found. Urban trees experience fluctuating environmental conditions (such as different pit characteristics, local traffic conditions, street infrastructure or the close proximity of buildings [63]) and simultaneously interact with a range of biota (such as bacteria or pathogenic fungi), which may weaken the effect of mycorrhizal diversity on tree health and make it impossible to detect [33]. Additionally, next generation amplicon sequencing is a very sensitive technique that has initiated a boom in mycorrhizal fungal ecology research [64]. It is, however, possible that it detects also a broad range of non-functional mycorrhizal taxa, which may not contribute to urban tree health.

### Implications

Although urban trees provide many ecosystem services to the urban population, tree health and survival is often compromised in urban areas [14,15]. Our results show that soil organic matter explains significant variation in tree health indicators, indicating that urban planners should not overlook the importance of the soil quality and its water holding capacity for the health of urban trees. Soil organic matter promotes soil aggregate formation and porosity, thus indirectly the water availability of urban trees and potentially also the ecosystem services delivered by urban trees. The accumulation of organic matter in the soil, however, depends on the interaction between soil organic matter quantity and quality factors (such as organic acids, proteins, humic acids or lignin), the soil matrix properties (such as pore size, pH, electrical conductivity and texture) and characteristics of the microbial communities (such as size, functional diversity and community composition). More specifically, the balance between organic matter inputs and the rate of organic matter decomposition by microbial communities is critical for the accumulation of soil organic matter [65]. In urban areas, however, organic matter inputs from plants and animals are low to non-existing. Therefore, adding organic matter, which has been shown to improve tree survival and growth [66,67], may be necessary to maintain the quality and water holding capacity of urban soils. Further research should also include an inventory of other soil microbiota which may independently, or in interaction with ectomycorrhiza, mediate tree health in urban settings.

Given its multifunctional role in forest ecosystems, mycorrhizal diversity may also potentially contribute to urban tree health [17]. In this study, however, no relation between mycorrhizal diversity and urban tree health could be demonstrated. To increase our understanding of the potential benefits of mycorrhizal diversity *in situ*, we suggest that future studies manipulate mycorrhizal communities in the field to assess their role on tree health and the delivery of ecosystems services.

## Supporting information

S1 Table The dataset used for analysis containing the tree health indicators, soil chemical variables and mycorrhizal diversity variables. (XLSX)
